# Theoretical vs. empirical discriminability: the application of ROC methods to eyewitness identification

**DOI:** 10.1186/s41235-018-0093-8

**Published:** 2018-03-14

**Authors:** John T. Wixted, Laura Mickes

**Affiliations:** 1 Department of Psychology, University of California, San Diego, CA USA; 2 Department of Psychology, Royal Holloway, University of London, London, UK

**Keywords:** Eyewitness identification, ROC analysis, Sequential lineups, Simultaneous lineups, Discriminability

## Abstract

Receiver operating characteristic (ROC) analysis was introduced to the field of eyewitness identification 5 years ago. Since that time, it has been both influential and controversial, and the debate has raised an issue about measuring discriminability that is rarely considered. The issue concerns the distinction between empirical discriminability (measured by area under the ROC curve) vs. underlying/theoretical discriminability (measured by *d’* or variants of it). Under most circumstances, the two measures will agree about a difference between two conditions in terms of discriminability. However, it is possible for them to disagree, and that fact can lead to confusion about which condition actually yields higher discriminability. For example, if the two conditions have implications for real-world practice (e.g., a comparison of competing lineup formats), should a policymaker rely on the area-under-the-curve measure or the theory-based measure? Here, we illustrate the fact that a given empirical ROC yields as many underlying discriminability measures as there are theories that one is willing to take seriously. No matter which theory is correct, for practical purposes, the singular area-under-the-curve measure best identifies the diagnostically superior procedure. For that reason, area under the ROC curve informs policy in a way that underlying theoretical discriminability never can. At the same time, theoretical measures of discriminability are equally important, but for a different reason. Without an adequate theoretical understanding of the relevant task, the field will be in no position to enhance empirical discriminability.

## Significance Statement

In many fields, an important applied goal is to identify diagnostic procedures that maximize discriminability (e.g., that maximize the ability to discriminate between patients who do vs. do not have a disease or to discriminate between suspects who are vs. are not guilty). Receiver operating characteristic (ROC) analysis has long been used in applied fields to measure discriminability, but it was only recently introduced to the field of eyewitness identification. Despite being introduced only 5 years ago, ROC analysis was endorsed by a National Research Council committee as an improvement over prior evaluation practices, and ROC-based research has already had a major influence on real-world policies concerning eyewitness identification procedures. Nevertheless, it remains controversial among eyewitness identification researchers, and a central issue in the debate concerns the distinction between theoretical and empirical discriminability. An understanding of that distinction is important for both theoreticians and policymakers because the two measures need not agree. For theoreticians, theoretical discriminability (e.g., *d'*) is the measure of interest, but for policymakers, empirical discriminability (e.g., area under the ROC) is the measure of interest.

## Introduction

Plotting the receiver operating characteristic (ROC) is a graphical method of data analysis that has been used for decades to measure discriminability, but what is discriminability, exactly? A consideration of that question seems appropriate in light of an on-going debate over the utility of ROC analysis in one particular area of applied psychology. Although widely used throughout experimental psychology and in many applied fields beyond psychology, ROC analysis has been controversial – and also influential – in the field of eyewitness identification (Lampinen, 2016; Levi, 2016; Rotello & Chen, 2016; Smith, Wells, Lindsay & Penrod, 2017; Wells, Smith, & Smalarz, 2015; Wells, Smalarz, & Smith, 2015; Wixted & Mickes, 2015a, 2015b; Wixted, Mickes, Wetmore, Gronlund, & Neuschatz, 2017). That recent controversy has brought to the surface an important distinction that is the focus of this article, namely, the distinction between theoretical (i.e., “underlying”) discriminability and empirical discriminability.

In experimental psychology, theoretical discriminability typically refers to the degree to which unobservable memory or perceptual signals from two classes of repeatedly presented stimuli – which we shall refer to as *target* stimuli and *foil* stimuli – overlap in the brain of a participant. If those two distributions overlap completely, then discriminability is equal to zero. The less they overlap, the higher discriminability is said to be. Theoretical discriminability is measured by a statistic like *d'*, which is the standardized distance between the means of two underlying strength distributions that are assumed to be Gaussian in form and to have equal variance. If a different model is assumed – even a slight variant that merely assumes unequal variances – then a different measure of discriminability would apply, such as *d*_*a*_ (see Macmillan & Creelman, 2005). Despite their differences, these alternative measures of theoretical discriminability will ordinarily agree about which of two conditions yields higher discriminability. However, that will not always be the case, and the fact that *d'* and *d*_*a*_ can disagree (e.g., see Dougal & Rotello, 2007) underscores the critical point that theoretical discriminability exists in relation to the model used to quantify it.

Empirical discriminability is not the same as theoretical discriminability. In particular, empirical discriminability does not refer to the separation between two unobservable distributions of memory or perceptual signals that occur in the brains of participants across target and foil trials. Instead, empirical discriminability refers to the degree to which participants correctly sort target and foil stimuli into their true categories. If the target and foil stimuli are both sorted into the “target” category with the same probability (as would happen if responding were random), then empirical discriminability would be equal to zero. The more the target stimuli are correctly placed into the target category and the foil stimuli are correctly placed into the “foil” category, the higher empirical discriminability is said to be. Empirical discriminability is measured using a non-parametric statistic known as area under the ROC curve (AUC). This measure is purely geometric and relies on no theoretical assumptions about the strengths of underlying memory signals. Thus, in contrast to theoretical discriminability, a non-parametric measure of empirical discriminability remains unchanged even if a new model of underlying discriminability is adopted.

In practice, just as different model-based measures of theoretical discriminability usually agree about whether discriminability is higher in Condition A compared to Condition B, so too do theoretical and empirical measures of discriminability usually agree about which condition is diagnostically superior (Mickes, Moreland, Clark, & Wixted, 2014; Rotello & Chen, 2016; Wixted et al., 2017). However, they need not agree, and that fact lies at the heart of the controversy over ROC analysis (Lampinen, 2016; Smith, Wells, Lindsay, & Penrod, 2017). In fact, as illustrated later, an empirical AUC measure can indicate that Condition A yields higher discriminability than Condition B even when a theoretical *d'* measure of underlying memory signals indicates the opposite.

What are the implications of the fact that theoretical and empirical measures of discriminability are capable of yielding conclusions that point in opposite directions? Basic and applied researchers alike may find it instructive to consider this issue, especially as it relates to use-inspired basic research. Such research is often focused on testing theories that may have applied significance. In such a study, which measure should be used if they happen to disagree about which of two conditions yields higher discriminability, a model-based theoretical measure like *d'* or a model-free empirical measure like AUC? Could both measures be right even when they reach opposite conclusions? And what would the policy implications be in a case like that?

Our claim is that both measures can, in fact, be right even when they reach opposite conclusions. They can both be right because they measure different aspects of memory. One measures the degree to which latent memory signals theoretically overlap in the brains of participants; the other measures the degree to which participants can use their memory to empirically sort innocent and guilty suspects into their true categories. Critically, when testing theoretical models, a theoretical measure of discriminability takes precedence, but when deriving real-world policy implications from the results, the empirical AUC measure of discriminability takes precedence. That is the main take-home message of this article. We consider this issue in relation to the empirical evaluation of competing lineup procedures (namely, simultaneous vs. sequential lineups) because it is ground zero of the recent controversy over the use of ROC analysis in the field of eyewitness identification. However, as we explain in more detail later, the same point applies to any discrimination procedure that has applied implications. Whether the policy decision involves eyewitness memory, medical diagnosis, or lie detection (to name a few applied fields), empirical discriminability takes precedence over theoretical discriminability.

## The basic signal detection framework for eyewitness identification procedures

In the course of a criminal investigation, the police will often identify a suspect – one who may or may not be guilty – and then rely on an eyewitness to help them determine if they have the right person. To do so, the police present the eyewitness with a recognition memory test. The police would like to use a recognition procedure that maximizes the chances that a guilty suspect will be identified (i.e., that maximizes the hit rate, HR) while minimizing the chances that an innocent suspect will be misidentified (i.e., while minimizing the false alarm rate, or FAR). In other words, the police face a signal detection problem, which is an issue that experimental psychologists literally wrote a book about (Green & Swets, 1966). Only recently, however, has signal detection theory been brought to bear on this issue.

**Fig. 1:**
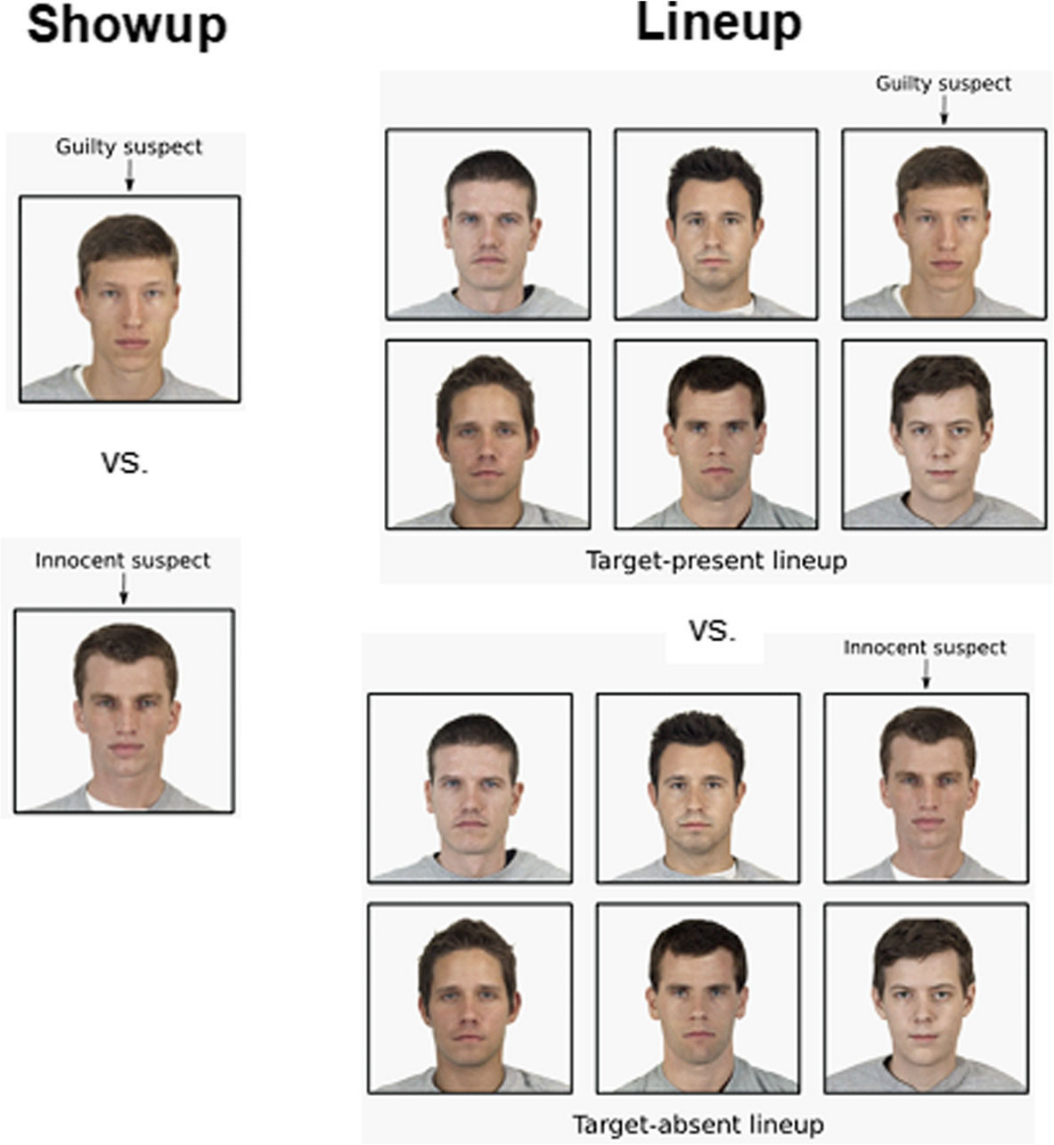
An illustration of two common eyewitness identification procedures. The left panel illustrates a showup in which the recognition memory test consists of a single photo – either the guilty suspect (the target) or an innocent suspect (the foil) – presented for a yes/no decision. The right panel illustrates a simultaneous lineup in which the recognition memory test consists of the presentation of a target-present array containing one guilty suspect (the target) and five fillers (foils) or a target-absent array containing one innocent suspect and five fillers (all foils). Suspect faces and filler faces from the Chicago Face database (Ma, Correll, & Wittenbrink, 2015)

One common eyewitness identification procedure is known as a showup, which is illustrated in the left panel of Fig. 1. In a showup, the eyewitness is presented with only one individual, who is either innocent or guilty. Usually, a police showup involves a live individual (not a photo) because it is used when a suspect is apprehended in the minutes following a crime and is then brought to the eyewitness to determine if that suspect is the perpetrator. Because a showup involves a single individual, it corresponds to what is commonly referred to as an old/new recognition memory task.

Another commonly used eyewitness identification task is a lineup, which is illustrated in the right panel of Fig. 1. In the US, the police test eyewitness memory with lineups hundreds of thousands of times every year. Live lineups were once the norm, but nowadays, the police almost always administer photo lineups after they identify a suspect in the days or weeks following a crime. A photo lineup consists of a picture of one suspect (the person who the police believe may have committed the crime) plus several additional photos of physically similar foils (i.e., fillers) who are known to be innocent. As illustrated in the right panel of Fig. 1, a target-present lineup includes the perpetrator along with (usually five) similar-appearing foils; a target-absent lineup is the same except that the perpetrator is replaced by an innocent suspect. The police do not know if they have constructed a target-present or a target-absent lineup, but if the eyewitness picks the suspect (innocent or guilty) it increases their confidence that they have found the perpetrator.

**Fig. 2:**
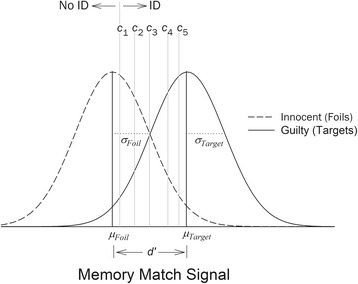
Equal-variance Gaussian signal detection model for a showup or a lineup. For a showup, the model operates in the same way that it does for a standard old/new recognition memory test. For a lineup, the simplest decision rule holds that a positive identification (ID) is made if the memory-strength of the strongest item in the array (considered in isolation) exceeds criterion, *c*_1_. In that case, the confidence rating associated with the ID depends on the highest confidence criterion that is exceeded (e.g., the confidence rating is 5 if the strength of the most familiar face exceeds *c*_5_)

A signal detection interpretation of showup performance is straightforward (Fig. 2). According to this model, the memory match signals generated by targets (guilty suspects) and foils (innocent suspects) are distributed according to Gaussian distributions with means of *μ*_*Target*_ and *μ*_*Foil*_, respectively, and standard deviations of *σ*_*Target*_ and *σ*_*Foil*_, respectively. The model depicted in Fig. 2 is an equal-variance model such that *σ*_*Target*_ = *σ*_*Foil*_, though one need not make that assumption. The target mean is higher than the foil mean because the target actually does correspond to the eyewitness’s memory of the perpetrator. The memory signals for both targets and foils vary from suspect to suspect because some guilty suspects (targets) are encoded better than others, and some innocent suspects (foils) will happen to coincidentally match the memory of target better than others. The difference between the target and foil means in terms of their common standard deviation is the main signal-detection-based measure of discriminability, *d'*.

Unless *d'* is very large, the two distributions overlap to some degree, which means that no signal strength perfectly distinguishes targets from foils. Thus, a decision criterion must be set such that any memory signal that exceeds it yields a positive identification (ID), whereas any memory signal that falls below it results in a non-identification (No ID). The HR corresponds to the proportion of the target distribution that exceeds the decision criterion, and the FAR corresponds to the proportion of the foil distribution that exceeds the decision criterion.

Confidence in an ID corresponds to the highest confidence criterion exceeded by the memory strength associated with a given face, whether it is a target or a foil. The model shown in Fig. 2 assumes that a 5-point confidence scale was used for an ID (1 = low confidence ➔ 5 = high confidence), and each confidence rating is associated with its own decision criterion. For example, a face that generates a memory signal that falls to the extreme right of the horizontal memory-strength axis will not only be identified but will be identified with high confidence. That is, because the memory signal falls above *c*_5_, the ID will be given a rating of 5 on the 5-point confidence scale.

The model shown in Fig. 2 applies directly to a recognition test in which the eyewitness is presented with a single test face, either a target (guilty suspect) or a foil (innocent suspect). The same basic model applies to a lineup, but the way it works is somewhat different. Basically, a six-item target-present lineup is conceptualized as five random draws from the foil distribution and one random draw from the target distribution; a six-item target-absent lineup is conceptualized as six random draws from the foil distribution (all statistically independent of each other in the simplest case). Note that the memory strength of the innocent suspect is represented by one of the values drawn from the foil distribution because, if the lineup is constructed in such a way that the innocent suspect does not stand out (i.e., if the lineup is fair), the innocent suspect is, from the point of view of the witness, just another individual who fits the description of the perpetrator but who did not actually commit the crime (i.e., the innocent suspect is just another foil).

Because multiple faces are involved on a given lineup test, more than one face may exceed the decision criterion. How does the witness decide whether or not to make an ID? The simplest decision strategy on both target-present and target-absent trials would be for the witness to first determine the photo that generates the strongest (MAX) signal and to then identify that person (who is either the suspect or a foil) if the signal exceeds a decision criterion. That person would be identified even if one or more of the other faces in the lineup also generate a memory signal that exceeds the decision criterion. If no face in the lineup generates a memory signal that exceeds the criterion, the lineup would be rejected (i.e., no ID would be made).

Although experimental psychologists have conceptualized and analyzed basic list memory performance in terms of signal detection theory for decades, for many years, lineup performance was analyzed without any guidance from that theory. We next review how lineup performance was originally analyzed and then consider how the basic model illustrated in Fig. 2 points to a better method of analysis, namely, ROC analysis. However, it is important to emphasize at the outset that ROC analysis is not inherently dependent on any theoretical consideration, including signal detection theory. A model like the one depicted in Fig. 2 guides thinking about why ROC analysis is important, but once that point is appreciated, ROC data can be collected and analyzed in a purely empirical way (i.e., without embracing any theoretical assumptions). A purely empirical ROC-based analysis of discriminability is what provides policymakers with the information they need to determine which eyewitness identification procedure is superior to another. After presenting that case, we go on to argue that, for theoretical purposes, ROC data from lineups can also be productively analyzed in a theory-dependent way using signal detection models like the one shown in Fig. 2.

## Simultaneous vs. sequential lineups

As noted above, a simultaneous photo lineup involves the simultaneous presentation of all of the faces in the lineup (Fig. 1). In a sequential lineup, the photos are instead presented one at a time. In most experimental studies of the sequential lineup, a stopping rule is used such that the first photo that elicits a “yes” response terminates the procedure. A considerable body of research has been interpreted to mean that sequential lineups, developed by Lindsay and Wells (1985), are diagnostically superior to simultaneous lineups (e.g., Steblay, Dysart, & Wells, 2011). Moreover, in terms of real-world impact, this line of research ranks among the most influential in all of experimental psychology. For example, a survey conducted in 2013 revealed that of more than 15,000 law enforcement agencies in the US, 30% had changed their policies and retrained their officers to administer the photo in a lineup sequentially instead of, or as an alternative option to, administering them simultaneously (Police Executive Research Forum, 2013).

The idea that sequential lineups might be superior to simultaneous lineups is based primarily on the results of mock-crime laboratory experiments conducted over the last 30 years. In a typical mock-crime experiment, participants witness a staged crime (e.g., by watching a video of someone snatching a purse) and are later shown a lineup in which the perpetrator is either present or absent, as illustrated in the right panel of Fig. 1. The job of the witness is to indicate whether the perpetrator (i.e., the “target”) is present in the photo array and, if so, to specify the target’s photo. On target-present trials, the witness can correctly identify the target, incorrectly identify a filler (i.e., a “foil”), or incorrectly reject the array. On target-absent trials, the witness can incorrectly identify the innocent suspect, incorrectly identify a filler, or correctly reject the array. This general experimental design has also been applied to certain visual search tasks (Cameron, Tai, Eckstein, & Carrasco, 2004; Michel & Geisler, 2011; Shaw, 1980; Swensson & Judy, 1981) and to some radiologic assessment tasks (Starr, Metz, Lusted, & Goodenough, 1975; Swensson, 1996).

Although a lineup is a somewhat complex recognition memory procedure compared to a showup, the relevant measure of accuracy is still based on some combination of the HR and the FAR. However, computing these measures for a lineup is not as straightforward as it is for the showup. For a lineup, the HR is the proportion of target-present lineups that resulted in a correct ID of the guilty suspect. For example, if 70% of target-present lineups resulted in a correct ID of the guilty suspect, 20% resulted in an incorrect ID of a filler, and 10% resulted in no ID, the HR would be .70. The FAR is the proportion of target-absent lineups that resulted in an incorrect ID of the innocent suspect. For example, if 6% of target-absent lineups resulted in an incorrect ID of the innocent suspect, 30% resulted in an incorrect ID of a filler, and 64% resulted in no ID, the FAR would be .06. The FAR is .06 and not .36 (.06 + .30) because, even though the 30% of misidentified fillers are “errors” in the context of a psychology experiment, in the context of a police lineup, they would never lead to a false conviction because the police know that the fillers are not guilty. Thus, filler IDs are relatively inconsequential errors and are, therefore, treated separately. To determine which lineup procedure is superior in an applied sense, the focus has always been placed on consequential suspect IDs (i.e., on the HR and FAR).

The original argument in favor of the sequential lineup procedure comes from combining the correct and incorrect suspect ID rates into a ratio known as the diagnosticity ratio (DR). More specifically, DR = HR/FAR. The DR is what is usually thought of as the likelihood ratio in the odds version of Bayes’ theorem, according to which the posterior odds of guilt are equal to the prior odds of guilt multiplied by the likelihood ratio. More formally, Bayes’ theorem compares the odds in favor of one hypothesis over another. The two hypotheses of interest here are:

- *H*_1_: the suspect is guilty
- *H*_2_: the suspect is innocent

Bayes’ theorem states that:

$$ \frac{P\left({H}_1|D\right)}{P\left({H}_2|D\right)}=\frac{P\left(D|{H}_1\right)}{P\left(D|{H}_2\right)}\frac{P\left({H}_1\right)}{P\left({H}_2\right)}, $$

where *D* is the data (a suspect ID in this case), *P*(*H*_1_|*D*)/*P*(*H*_2_|*D*) represents the posterior odds of *H*_1_ compared to *H*_2_ (i.e., the odds of guilt after a suspect ID has been made), *P*(*D*|*H*_1_)/*P*(*D*|*H*_2_) represents the likelihood ratio (i.e., the diagnosticity ratio) and *P*(*H*_1_)/*P*(*H*_2_) represents the prior odds of *H*_1_ compared to *H*_2_ (i.e., the odds of guilt before a suspect ID has been made).

In the lineup scenario, *P*(*D*|*H*_1_) is the HR (i.e., the correct ID rate) and *P*(*D*|*H*_2_) is the FAR (i.e., the false ID rate). Thus, the DR (i.e., the likelihood ratio) is equal to the correct ID rate divided by the false ID rate (HR/FAR). In most experiments, half the participants are presented with a target-present lineup and half are presented with a target-absent lineup, which means that the base rate of guilt equals the base rate of innocence. Under such conditions, the prior odds of guilt, *P*(*H*_1_)/*P*(*H*_2_), equal 1, in which case the DR directly indicates the posterior odds of guilt. For example, if the prior odds are equal to 1, and if the HR = .50 and the FAR = .10, the resulting DR of 5 would mean that a suspect identified using this procedure is five times as likely to be guilty as innocent. Note that this measure is computed only from witnesses who identify a suspect (i.e., witnesses who pick a filler or make no ID are not involved in the calculation). This makes sense because the question of primary interest concerns how to interpret the outcome that imperils a lineup member. Filler IDs do not (because, as noted earlier, fillers are known to be innocent) and neither do no IDs, but suspect IDs do, whether the identified suspect is innocent or guilty.

The posterior odds of guilt can, of course, also be computed for the other two outcomes, namely, filler IDs and lineup rejections (Wells, Yang, & Smalarz, 2015). For example, if the prior odds of guilt are even (i.e., half target-present lineups, half target-absent lineups), one can ask about the posterior odds of guilt for the subset of lineups that resulted in a filler ID or No ID. Given either of those outcomes, laboratory studies suggest that the posterior odds of guilt are slightly less than even (Wells, Smalarz, et al., 2015, Wells, Smith, et al., 2015, Wells, Yang, et al., 2015), which means that filler IDs and lineup rejections are slightly probative of innocence. However, our focus here, like most of the focus in the prior academic literature, is on the far more consequential outcome, suspect IDs (and the corresponding measures, namely, the HR and the FAR). In other words, our main focus is on the measures that once led the field to conclude that sequential lineups are diagnostically superior to simultaneous lineups.

In the seminal study on this issue, Lindsay and Wells (1985) reported that for the sequential lineup, HR = .50 and FAR = .17 (DR_SEQ_ = .50/.17 = 2.94), whereas for the simultaneous lineup, HR = .57 and FAR = .42 (DR_SIM_ = .57/.42 = 1.36). It seems fair to say that, to many, the large reduction in the FAR is what makes the sequential procedure so attractive. However, upon reflection, it becomes clear that one must consider the effect on the HR as well. At first, the relatively small decrease in the HR associated with switching to the sequential procedure seems reassuring. However, because this “hand waving” analysis of the effect of sequential lineups on the HR and FAR is clearly insufficient, a quantitative assessment of some kind is needed. The DR provides one way to quantify the effects of interest.

The DR is related to positive predictive value (PPV), which is the probability that a suspect who has been identified is actually guilty. The equation relating these two measures is as follows:

$$ PPV=\frac{\left[ DR\frac{P\left({H}_1\right)}{P\left({H}_2\right)}\right]}{\left[ DR\frac{P\left({H}_1\right)}{P\left({H}_2\right)}+1\right]}, $$

where, again, DR = *P*(*D*|*H*_1_)/*P*(*D*|*H*_2_) and *P*(*H*_1_)/*P*(*H*_2_) represents the prior odds of guilt. For the typical equal base-rate situation, where *P*(*H*_1_)/*P*(*H*_2_) = 1, this equation reduces to PPV = DR/(DR + 1). Thus, for the Lindsay and Wells (1985) study, PPV_SEQ_ = 2.94/(2.94 + 1) = .746 and PPV_SIM_ = 1.36/(1.36 + 1) = .576. In other words, the probability that a suspect identified from a sequential procedure is guilty is .746, whereas the probability that a suspect identified from a simultaneous procedure is guilty is .576. These PPV values are not very impressive for either procedure, but the task used by Lindsay and Wells (1985) involved an innocent suspect who closely resembled the perpetrator (i.e., it was designed to be a hard task). In the most recent meta-analysis of the academic literature, Steblay et al. (2011) argued that this pattern is fairly typical of studies conducted since 1985 even when overall performance is better. That pattern (DR_SEQ_ > DR_SIM_) is what is meant by a “sequential superiority effect.”

## Empirical discriminability

Although the DR is a purely empirical (i.e., non-theoretical) measure in that it appeals to no model of latent memory strengths, it does not measure empirical *discriminability*, which refers to the degree to which innocent and guilty suspects are correctly sorted into their true categories. More specifically, an empirical measure of discriminability quantifies the degree to which innocent suspects are correctly sorted into the “innocent” category while, at the same time, guilty suspects are correctly sorted into the “guilty” category. In this section we explain why the DR does not unambiguously identify the diagnostically superior procedure and why a non-theoretical empirical measure of discriminability instead provides the needed information to inform policy decisions. We use a signal detection model like the one shown in Fig. 2 to guide thinking about this issue, but, again, at this stage, it is no more than a conceptual guide. The empirical ROC analysis we describe is not dependent on any of the assumptions of that model. Later, we present a theoretical analysis of underlying discriminability that is dependent on those assumptions.

Like the DR, a measure of true empirical discriminability also makes use of the HR and FAR computed from IDs made to suspects in the lineup. Again, it often seems insufficient to assess the diagnostic accuracy of a lineup using a measure that includes only the HR and the FAR, which leaves out any consideration of filler IDs from target-present and target-absent lineups. However, unlike a filler, an innocent suspect is not known to be innocent and will be imperiled (and perhaps wrongfully convicted) if mistakenly identified. For that reason, a reasonable goal for the police is to maximize the HR while simultaneously minimizing the FAR (values that are computed using only suspect IDs), regardless of the rate of filler IDs. For example, if Procedure A yielded a HR of .80 and a FAR of .05, whereas Procedure B yielded a HR of .60 and a FAR of .20, it would be difficult to defend the argument that the police should use Procedure B based on some consideration having to do with the rate of filler IDs vs. no IDs for the two procedures. Instead, Procedure A would clearly be the one to use no matter what the filler ID rate and no ID rate happened to be for either procedure. Moreover, no model of memory would be needed to reinforce the decision as to which of the two procedures is diagnostically superior. On their own, the empirical data would make it crystal clear which procedure is diagnostically superior.

The example presented above involved an easy choice because Procedure A yielded *both* a higher HR and a lower FAR than Procedure B. However, the issue becomes more complicated when the HR and FAR are both higher for one procedure than the other. For example, which procedure would be diagnostically superior if Procedure A yielded a HR of .80 and a FAR of .05, whereas Procedure B yielded a HR of .60 and a FAR of .02? The DR for Procedure B (.60/.02 = 30) is higher than that for Procedure A (.80/.05 = 16), so by that measure Procedure B would be preferred. However, this outcome would not actually identify Procedure B as being diagnostically superior. Why not? In brief, the reason is that it would be easy to selectively induce more conservative responding for Procedure A (e.g., using instructions that encourage a high degree of certainty before making an ID from the lineup), thereby lowering both the HR and the FAR for that procedure. Imagine that when conservative responding was encouraged for Procedure A, the HR dropped from .80 to .65 (still higher than Procedure B, with a HR of .60) and the FAR dropped from .05 to .02 (now equal to Procedure B). Under these conditions, Procedure A would clearly be the diagnostically superior procedure. Instead of switching from Procedure A to Procedure B to achieve a FAR as low as .02, it would make more sense to stick with Procedure A and to induce more conservative responding. In essence, that kind of comparison is what ROC analysis is all about, and it illustrates why ROC analysis is needed to unambiguously determine the diagnostically superior procedure.

### ROC analysis

**Fig. 3:**
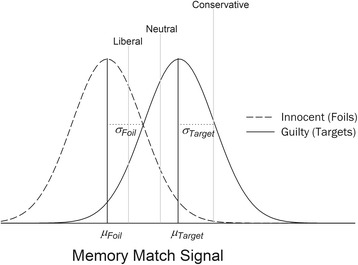
An equal-variance Gaussian signal detection model illustrating the placement of three different decision criteria (liberal, neutral and conservative)

ROC analysis begins with measuring an entire family of hit and false alarm rates for each diagnostic procedure, and there is more than one way to do so. For example, as mentioned above, different instructions can be used to manipulate response bias across different conditions (e.g., liberal, neutral and conservative). In the liberal response bias condition, the instructions would actively encourage participants to make an ID from the lineup, resulting in relatively high hit and false alarm rates, as illustrated in Fig. 3. Conceptualized in terms of signal detection theory, a liberal placement of the decision criterion results in a large proportion of the target distribution and a large proportion of the foil distribution exceeding it. In the conservative response bias condition, the instructions would *discourage* participants from making an ID unless a participant is quite certain of being correct (i.e., the decision criterion in Fig. 3 moves to the far right). These instructions would result in relatively low hit and false alarm rates. In a neutral response bias condition, the instructions would neither encourage nor discourage participants from making an ID, resulting in intermediate hit and false alarm rates. When the hit and false alarm rates from the different biasing conditions are plotted against each other on a graph (HRs on the *y*-axis, false alarm rates on the *x*-axis), they make up the instruction-based ROC.

A more common and arguably better method of generating ROC data – and the method that we will focus on here – makes use of confidence ratings that participants provide when they make an ID from a lineup. The confidence ratings themselves provide the multiple decision criteria needed to construct an ROC, as illustrated earlier in Fig. 2. Thus, only a single condition is needed in this case, one in which neutral response bias instructions would be used. A common neutral instruction uses words to this effect: “the perpetrator may or may not be in the lineup, and it is just as important to exonerate an innocent suspect as it is to identify the guilty suspect.” The first point on the confidence-based ROC is obtained by computing the HR and FAR in the usual way, namely, by counting all suspect IDs from target-present and target-absent lineups regardless of the confidence expressed by the participant. In terms of the model shown in Fig. 2, a suspect ID would be counted whenever the suspect generated the MAX signal in the lineup and the signal exceeded *c*_1_. This (most liberal) ROC point is associated with the highest HR and FAR for a given condition, and these are the values that have long been used to compute the DR (HR/FAR).

Additional (more conservative and, therefore, lower) hit and false alarm rates are computed by setting an ever-higher standard on the confidence scale for counting IDs. Thus, for example, the second ROC point is obtained by counting all suspect IDs *except* those that were made with the lowest level of confidence (i.e., by treating as a non-ID any suspect ID that is acknowledged by the participant to be little more than a guess). In terms of the model shown in Fig. 2, a suspect ID would be counted whenever the suspect generated the MAX signal in the lineup and the signal exceeded *c*_2_. The last ROC point is computed by counting only suspect IDs that were made with the highest level of confidence (i.e., for suspects with a MAX signal exceeding *c*_5_). This (most conservative) ROC point is associated with the lowest correct and false ID rates for a given condition. Mickes et al. (2017) recently compared the instruction-based and confidence-based methods of generating ROC data for lineups and found that they yielded similar (though not identical) curves.

The reason why it is arguably better to use neutral instructions plus confidence ratings to collect ROC data is that it allows a different decision criterion to be used at different stages of an investigation. Early in an investigation, it would make sense to use a relatively liberal criterion. If the witness identifies the suspect with low confidence, for example, the police may wish to further investigate that individual (e.g., by using that low-confidence ID to support a request for a search warrant from a judge). At a later stage of the investigation, however, it would make sense to set a much higher standard before indicting the identified suspect if that indictment is going to be based largely on the eyewitness identification evidence. If, instead of using confidence ratings, instructions were used to induce conservative responding from the outset such that only IDs made with high confidence were obtained in the first place, the police would lose the potentially useful investigatory information that a suspect ID made with low or medium confidence might provide.

### A hypothetical example

Table 1 presents hypothetical data that might be observed on a lineup task where confidence ratings were taken using a 5-point confidence scale. These hypothetical data might be from an individual observer, or they might reflect data aggregated across many observers, with the latter usually being true of eyewitness identification research in which each participant is usually tested only once (as is usually true of real eyewitnesses). For these hypothetical lineup data, confidence ratings were not collected when the photo array was rejected (No ID). This is typical of lineup experiments in which participants are asked how certain they are that the most familiar person in the lineup is the perpetrator when that person is identified, but they are not asked how certain they are that the most familiar person in the lineup is *not* the perpetrator when no one is identified. Often, they are asked to make a global confidence rating in a non-ID (e.g., “I am 80% certain that the perpetrator is not in the lineup”), but this approach does not provide enough information to compute additional hit and false alarm rates (i.e., additional ROC points) beyond those computed from positive IDs. To compute additional ROC points from non-IDs, the confidence rating would need to be specifically applied to the face that the witness believes is most likely to be the perpetrator (e.g., “Face #3 is most likely to be the perpetrator, but I am 80% certain that he is not the one”). To date, confidence ratings have not been collected in that manner.

**Table 1:** Hypothetical frequency counts of target positive identifications (IDs), foil IDs and No IDs by level of confidence for target-present and target-absent lineups

Confidence	Target-present			Target-absent	
	Target	Foil	No ID	Foil	No ID
1	34	30	191	57	330
2	33	24		41	
3	30	17		27	
4	36	16		23	
5	74	17		22	

**Fig. 4:**
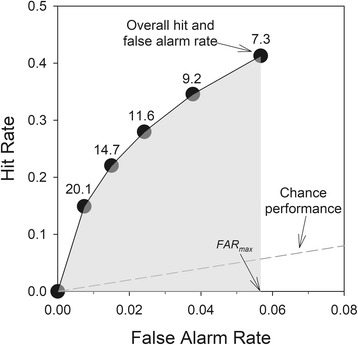
Hypothetical receiver operating characteristics (ROC) curve for a lineup procedure in which a 5-point confidence scale was used. The number above each point is the diagnosticity ratio for that correct and false positive identification (ID) rate pair. The region shaded in light gray represents the partial area under the ROC curve (pAUC) for the specified false ID rate range of 0 to *FAR*_*max*_, which is equal to .057 in this case. The diagonal line represents chance performance (where correct ID rate = false ID rate)

The data in Table 1 allow one to compute not only the overall HR and FAR but also a HR and FAR separately for varying degrees of response bias specified by the different confidence ratings. Those data constitute the ROC for a given lineup procedure. Figure 4 presents the ROC data computed from the values shown in Table 1. The HR for each level of confidence is computed by counting the number of correct (i.e., target) IDs made from target-present lineups with that level of confidence or a higher level of confidence, divided by the total number of target-present lineups. The false alarm rate for each level of confidence is computed by counting the number of foil IDs made from target-absent lineups with that level of confidence or a higher level of confidence, divided by the total number of target-absent lineups and divided again by lineup size (6). Only one of the foils fills the role of the innocent suspect, which is why the value is divided by lineup size. Doing so yields an estimate of the false *suspect* ID rate (i.e., the FAR). Essentially the same estimate would be obtained if one of the foils had been pre-designated to serve as the innocent suspect (as in Fig. 1) and then only IDs to that face were counted as false alarms.

### Measuring the area under the ROC curve

To estimate *empirical* discriminability (i.e., to measure the ability of participants to correctly sort innocent and guilty suspects into their true categories), an area under the curve measure is computed. As noted above, lineup ROCs are usually based only on positive IDs (positive Target IDs from target-present lineups and positive foil IDs from target-absent lineups), which yields a truncated, partial ROC compared to the ROCs obtained from non-lineup tasks. In non-lineup tasks, the FAR typically ranges from 0 to 1, but in a fair lineup ROC, it only ranges from 0 to 1/6 (.167). That is the maximum possible FAR because even if every witness presented with a fair target-absent lineup identified someone (i.e., if responding were maximally liberal such that a “yes” response were made to every target-absent lineup), witnesses would land on the innocent suspect by chance only 1/6 of the time and would land on a filler the other 5/6 of the time. In actual practice, the obtained FAR is typically much less than .167 (as it is in Fig. 4) because, typically, responding is not maximally liberal.

A similar (but not identical) story applies to the HR. In non-lineup tasks, the HR typically ranges from 0 to 1. However, in a fair lineup ROC, it usually ranges from 0 to a value less than 1, such as .80. The reason is that even if every participant presented with a target-present lineup identified someone (i.e., if responding were maximally liberal), it is unlikely that everyone would successfully recognize the guilty suspect because it is unlikely that everyone formed a clear memory of the perpetrator at study. Therefore, under typical imperfect memory conditions, some participants would land on a target-present filler, in which case the maximum HR would be less than 1. The HR will reach 1.0 only when every participant forms a clear enough memory of the perpetrator to identify that individual from a target-present lineup.

Because the FAR for a lineup is limited to a range that is less than 0 to 1, the relevant measure of empirical discriminability for a lineup is the *partial* area under the curve (pAUC). The partial area under the curve is computed from a false alarm rate of 0 up to some maximum that is less than or equal to .167.^1^ That maximum FAR is denoted here as *FAR*_*max*_. An obvious choice for *FAR*_*max*_ is the FAR associated with the overall hit and false alarm rate that corresponds to the rightmost ROC point (.413 and .057, respectively, in Fig. 4). With *FAR*_*max*_ = .057, as it is in Fig. 4, pAUC for these data (i.e., the area of the shaded region) is approximately .017. An intuitive appreciation of why the value is approximately .017 can be obtained by considering the rectangle formed by the FAR and HR, with the short side of the rectangle defined by the FAR range from 0 to .057 and the long side defined by the HR range from 0 to .413. The area of that rectangle is equal to .057 × .413 = .024. The shaded area is a bit more than half of the area of that rectangle and is, therefore, equal to approximately .017.

This empirical measure of discriminability is not based on any theoretical assumptions about memory. In fact, no part of the ROC analysis presented thus far depends on any theoretical assumptions about underlying (latent) memory strengths. First, the hit and false alarm rates comprising the ROC were computed directly from the data (consisting of confidence-based frequency counts) and then plotted against each other. Next, the points were connected by straight lines. Finally, the area beneath the curve was estimated from a FAR of 0 to *FAR*_*max*_. Computer software is needed to precisely measure the size of the shaded area, and the tutorial videos associated with Gronlund, Wixted, and Mickes (2014) explain how to use one such R program, called pROC (Robin et al., 2011), to do that. The key point is that the program does not make any theoretical assumptions about latent memory strength signals to estimate the partial area under the curve (pAUC). Instead, it measures the shaded area in Fig. 4 atheoretically.

**Fig. 5:**
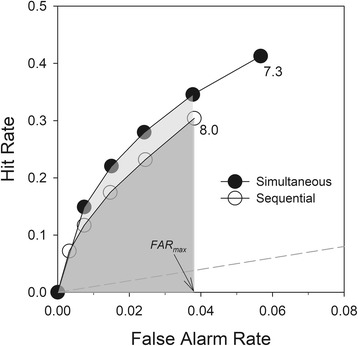
Hypothetical receiver operating characteristic (ROC) curves for two eyewitness identification procedures in which a 5-point confidence scale was used. The rightmost ROC point again represents the overall correct and false positive identification (ID) rates that are ordinarily used to compute the diagnosticity ratio. Note that the diagnosticity ratio for the rightmost point is higher for the sequential procedure, a result that, in the past, would have been interpreted to mean that the sequential procedure is diagnostically superior to the simultaneous procedure. The region shaded dark gray represents the partial area under the curve (pAUC) for the sequential procedure in the specified false ID rate range of 0 to *FAR*_*max*_. That dark gray region plus the light gray region above it represents the pAUC for the simultaneous procedure over the same false ID rate range. The dashed line represents the line of chance performance

What does the pAUC measure actually tell you? On its own, not very much. However, the usual goal is to compare the pAUC values for two different lineup procedures. That comparative analysis is extremely informative because the procedure that yields the higher pAUC is the diagnostically superior procedure. A practical consideration that arises in such an analysis is that the two procedures will not typically yield the same maximum FAR (i.e., the FARs with their respective rightmost ROC points). To compare the two procedures with respect to pAUC, it is essential to use the same *FAR*_*max*_ to measure the area under both curves. Which FAR – the one associated with the less conservative procedure or the one associated with the more conservative procedure – should determine the *FAR*_*max*_ used to compute pAUC for both procedures? To avoid any theoretical extrapolation of the ROC curve, it makes sense to set *FAR*_*max*_ equal to the FAR associated with the rightmost point of the more conservative of the two procedures being compared, as illustrated using hypothetical data in Fig. 5. Such an analysis covers a range that includes empirical ROC data generated by both procedures and, therefore, does not involve theoretically extrapolating the ROC curve to the right for either procedure.

A stickler might contend that a *minimum* FAR greater than 0 should also be specified, one that is equal to the FAR associated with the leftmost ROC point from the condition with the larger minimum FAR (e.g., *FAR*_*min*_ would be set to the FAR associated with the leftmost ROC point for the simultaneous procedure in Fig. 5). This approach would avoid extrapolating the ROC curve to the origin (0,0). However, in practice, *FAR*_*min*_ is usually set to 0 because no specific theory is relied upon to justify the seemingly safe assumption that if responding were infinitely conservative, both the HR and the FAR would be 0.

Once the minimum and maximum FAR values are specified, the pAUC measure for each procedure is fixed and will not vary as a function of which theoretical model of memory strengths is assumed to be true. In that sense, pAUC is a purely empirical measure of discriminability. In Fig. 5, it is visually obvious that pAUC for the simultaneous procedure is greater than the pAUC for the sequential procedure over the FAR range of 0 to .038 (the maximum FAR for the sequential procedure). This is true even though, as ordinarily computed, the DR for the sequential procedure (8.0) is larger than the DR for the simultaneous procedure (7.3). The pROC software uses a bootstrap procedure to determine if the apparent difference in the two pAUC values is statistically significant.

Assume that the difference is significant. What would it mean that pAUC_SIM_ > pAUC_SEQ_? This is the key question. It would mean that for any point on the lower (sequential) ROC that might be preferred, there is a point that can be generated by the other (simultaneous) procedure that has the same FAR and a higher HR. Imagine, for example, that policymakers were satisfied with the FAR associated with the rightmost point on the sequential ROC (.038). It is visually apparent that the simultaneous procedure can achieve that same FAR but with a higher HR. For a FAR of .038, the HR for the sequential procedure is .304, but the HR for the simultaneous procedure is .346. Moving slightly to the left on the simultaneous ROC would yield a HR that still exceeds .304 and that has a FAR of less than .038. Thus, the fact that that pAUC_SIM_ > pAUC_SEQ_ means that the simultaneous procedure can achieve *both* a higher HR *and* a lower FAR than the sequential procedure, at least in the FAR range of 0 to .038. No theoretical considerations are needed to appreciate the fact that results like these would establish that the simultaneous procedure is diagnostically superior to the sequential procedure in that FAR range.^2^

Then again, this analysis would not conclusively establish that the simultaneous procedure is necessarily superior outside of the tested FAR range (i.e., outside of 0 to *FAR*_*max*_). For some policymakers, the ideal FAR might fall outside of the tested range. For a given procedure, the ideal point on the ROC is, in part, a function of the subjective values associated with hits, false alarms, correct rejections, and misses (see Equation 1.14 in Green & Swets, 1966, p. 22). Because subjective values are involved, science cannot conclusively specify the point on the ROC that yields the highest utility. Science *can* conclusively specify the procedure that yields the highest ROC, but choosing the appropriate tradeoff between hits and false alarms on a given ROC is a matter for policymakers to decide. If, for some reason, policymakers preferred a FAR of approximately .06 because of the higher HR that could be achieved, the fact that pAUC_SIM_ > pAUC_SEQ_ over the tested FAR range (0 to *FAR*_*max*_ = .038) would not necessarily indicate that the simultaneous procedure is superior in a higher FAR range.

To find out if pAUC_SIM_ > pAUC_SEQ_ over a higher FAR range, one could use instructions to induce more liberal responding for the sequential procedure so that its maximum FAR also approaches .06. Alternatively, as noted earlier, confidence in No IDs could be collected in such a way as to allow one to project the ROC further to the right (i.e., by collecting a confidence rating in connection with the face that the witness believes is most likely to be the perpetrator). Looking at the two ROC curves in Fig. 5 and mentally projecting the sequential ROC curve to the right, it seems fairly safe to assume it would still fall below the simultaneous ROC. Nevertheless, to be sure about that, one would have to actually perform the experiment because it is at least theoretically possible that the ROC curves would cross and the sequential procedure would become superior in that higher FAR range. Despite that theoretical possibility, in the FAR range covered by this analysis (0 to .038), data like these would indicate a simultaneous superiority effect.

As a general rule, eyewitness ID researchers have been most interested in determining which procedure yields superior diagnostic performance over a range in which even *FAR*_*max*_ is low, thereby keeping the risk of falsely identifying an innocent suspect low. To date, the empirical ROC analyses that have been performed unanimously suggest that the simultaneous procedure yields a higher pAUC than the sequential procedure (Carlson & Carlson, 2014; Dobolyi & Dodson, 2013; Gronlund et al., 2012; Exp. 1 of Mickes, Flowe, & Wixted, 2012). Other studies have reported no significant difference between the two procedures, but with a trend still favoring the simultaneous procedure (e.g., Andersen, Carlson, Carlson, & Gronlund, 2014; Exp. 1b and Exp. 2 of Mickes et al., 2012; Terrell, Baggett, Dasse, & Malavanti, 2017). Moreover, two police department field studies subsequently reported findings consistent with the results of these laboratory studies (Amendola & Wixted, 2015, 2017; Wixted, Mickes, Dunn, Clark & W. Wells, 2016). Should these finding hold in future investigations, they would reverse the conclusions of DR-based psychological research that convinced 30% of US law enforcement agencies to adopt the sequential lineup procedure.

### Policy changes despite the controversy

The on-going debate over the utility of ROC analysis applied to eyewitness identification procedures has not greatly limited either its scientific impact or its real-world impact. In 2014, the National Academy of Sciences (NAS) convened a committee to evaluate the science of eyewitness identification. One focus of their work was to adjudicate the debate over whether the diagnosticity ratio or ROC analysis offers the best approach for comparing competing eyewitness identification procedures. With regard to relative merits of ROC analysis vs. the diagnosticity ratio, they came to the following conclusion:

Perhaps the greatest practical benefit of recent debate over the utility of different lineup procedures is that it has opened the door to a broader consideration of methods for evaluating and enhancing eyewitness identification performance. ROC analysis is a positive and promising step with numerous advantages. For example, the area under the ROC curve is a single-number index of discriminability (National Research Council, 2014, p. 86).

In light of that development, substantial changes have been made in terms of policy in law enforcement. A driving force behind the adoption of the sequential lineup procedure by many police departments was a policy adopted by the International Association of Chiefs of Police (IACP) in 2006, which encouraged sequential administration of both photo and live lineups (International Association of Chiefs of Police, 2006). However, in September of 2016, the IACP dropped its longstanding recommendation in favor of the sequential procedure. Their current model policy states: “This policy recognizes that the sequential and simultaneous approaches are both valid methods of conducting an identification procedure and does not recommend one over the other.” (International Association of Chiefs of Police, 2016, p. 1).

More recently, on 6 January 2017, the Department of Justice (DOJ) released a memo to federal prosecutors and federal law enforcement agencies concerning procedures for conducting photo lineups (Yates, 2017). The memo noted that research and practice had evolved significantly since the DOJ last addressed eyewitness identification issues in 1999 and stated that “…there has been an evolution in views on whether the ‘sequential’ administration of a photo array (presenting the witness one photo at a time) results in more accurate identifications than a ‘simultaneous’ administration (presenting all of the photos at once)” (p. 1) and went on to stress that administrators may use either simultaneous or sequential photo arrays. An appendix to the memo noted that recent research has suggested that “… simultaneous procedures may result in more true identifications and fewer false ones,” (p. 8) which is a succinct summary of what a procedure that yields a higher ROC can achieve. These policy changes were based on an AUC measure (specifically, pAUC), not a theory-based measure of discriminability like *d'*.

## Parametric analyses of AUC

The idea that policy is informed by an AUC measure of discriminability appears to be generally accepted in other applied fields, such as radiology, diagnostic medicine, and polygraph lie detection. In radiology, for example, Gallas et al. (2012) noted that “The paradigm of ROC analysis, and the measurement of the AUC in particular, is essential to the field of diagnostic imaging assessment” (p. 466). Similarly, when comparing the usefulness of different biomarkers for diagnosing prostate cancer, a recent review of the academic literature noted that “…the most common analysis, by far, is the area under the receiver-operating characteristics curve” (Evaluation of Genomic Applications in Practice and Prevention (EGAPP) Working Group, 2014, p. 341). And in a review of polygraph lie detection research, the National Research Council (2003) stated that “We used the area under an ROC curve extrapolated from each dataset to summarize polygraph accuracy as manifested in that dataset” (p. 342). Thus, our suggestion that policy decisions are informed by area under the ROC, not by a theoretical estimate of underlying discriminability, is new to the field of eyewitness identification but is not a new suggestion generally speaking.

Although, ideally, AUC would be measured non-parametrically – that is, without relying on any assumptions that might be wrong – a Gaussian model is sometimes used to measure it parametrically. For example, in their review of polygraph testing, the National Research Council (2003) used an equal-variance Gaussian model to measure *A*_*z*_ (a parametric measure of AUC) for studies that reported only a single point on the ROC. When no more than a single ROC point is available, the only way to obtain a non-parametric measure of the AUC would be to draw two lines extending from that point – one to the lower left corner of the ROC and the other to the upper right corner – and to then compute the area beneath the resulting polygon. Because, in practice, empirical ROC data are almost always curvilinear, this approach would likely underestimate the true AUC. To address that limitation, a Gaussian model can be used to more realistically extrapolate the empirical ROC curve so that the AUC can be measured parametrically (Macmillan & Creelman, 2005).

The Gaussian model that is used to extrapolate the empirical ROC curve and to then compute the area beneath it looks much like the signal detection model of memory depicted in Fig. 2 (e.g., two Gaussian distributions separated by *d'*). However, unlike Fig. 2, when using a Gaussian model for this purpose, the nature of the variable represented on the *x*-axis is not a relevant consideration. Instead, the *x*-axis represents whatever underlying variables might combine to determine empirical performance. The only assumption that this model makes is a statistical one, namely, that the aggregate underlying variable, whatever it might be, can be adequately modeled by two Gaussian distributions. That token model is then used to fit a curve through the one available ROC data point. Once the empirical trajectory of the ROC curve is extrapolated in that manner, the area beneath it (i.e., the measure of interest for policymaking purposes) can be computed.

A similar strategy has been recommended in the field of eyewitness identification when the data are such that ROC analysis cannot otherwise be performed. Specifically, Mickes et al. (2014) recommended that empirical discriminability be estimated by computing Gaussian-based *d'* for lineup studies that report only a single ROC point. Estimating *d'* (specifically, z-transformed HR minus z-transformed FAR) from a single ROC point is analogous to estimating *A*_*Z*_ from a single ROC point because *d'* and *A*_*Z*_ are monotonically related by the following equation (Macmillan & Creelman, 2005):

$$ {A}_Z=\Phi \left({d}^{\prime }/\sqrt{2}\right). $$

Thus, our claim that policymakers care only about AUC (not a theory-based measure of *d'*) should not be construed as an indictment of the use of a generic Gaussian-based statistical model to parametrically estimate AUC (or pAUC in the case of lineups) when such an estimate could not otherwise be obtained.

Unlike a policymaker, a theoretician is interested in estimating the specific underlying variables that affect empirical discriminability. For example, empirical ROC data will decrease toward the diagonal line of chance performance the more that (1) the memory signals for targets and foils overlap and/or (2) the confidence criteria vary from decision to decision (Wickelgren & Norman, 1966). Area under the curve measures (both non-parametric pAUC and parametric *A*_*Z*_) will also decrease towards their minimum values of .50 in either case. But a theory of lineup memory would make a specific prediction about the first latent variable (the distribution of memory signals) without necessarily making any prediction about the second (criterion variability). In parametric ROC analysis, the effects of those latent variables are purposefully conflated because the only goal is to obtain a “best guess” as to the likely empirical trajectory of the ROC curve (had those empirical ROC data been collected). To test the predictions of a theory of memory, a statistical model like that must give way to a model of memory signals that can measure the latent variables of interest. We turn to that issue next.

## Theoretical models of latent variables

A signal-detection-based theory about how the photos in a lineup generate memory signals makes a prediction about the degree to which *memory signals* for targets and foils overlap. We might refer to that memory-based *d'* as *d'*_*m*_, where the subscript *m* stands for “memory.” As noted by Wickelgren and Norman (1966), when *d'* is computed from the HR and FAR of an old/new recognition procedure, different sources of variance (e.g., variability in memory signals and variability in criterion placement) are conflated. If one assumes that there is no criterion variability, then *d'* = *d'*_*m*_. More realistically, criterion variability is assumed to exist, but its effects are usually (at least implicitly) assumed to be small and to be equal across two conditions. Under those assumptions, *d'* can be safely compared for Condition A vs. Condition B to test theory-based predictions about *d'*_*m*_. That is, if *d'* differs significantly across conditions, the difference can be attributed to a difference in *d'*_*m*_ despite the presumed existence of some criterion variability. This is how performance is usually assessed in studies of recognition memory (i.e., *d'* is measured for each condition, and any difference is usually attributed to a difference in the degree to which memory signals overlap).

As described above, because an AUC measure is affected by latent variables other than just *d'*_*m*_, the two measures are potentially dissociable. But there is more to the story of their potential dissociability because even when all latent variables are equated for two procedures, AUC and *d'*_*m*_ can *still* disagree. In this regard, it has long been known that *d'*_*m*_ may not directly correspond to AUC when comparing old/new vs. 2-alternative forced-choice (2AFC) recognition memory. For the old/new task, the participant is assumed to say “old” if the test item exceeds a decision criterion and to say “new” otherwise. For the 2AFC task, the simplest strategy would be for the participant to simply choose the more familiar of the two test items.^3^ In that case, for both test formats, the memory signals are drawn from the same distributions, so *d'*_*m*_ is the same in both cases. Yet, due to structural differences in the testing procedures, the empirical HR-FAR pair obtained from the 2AFC procedure will fall on a higher ROC curve than the HR-FAR pair obtained from old/new recognition (Macmillan & Creelman, 2005). If an atheoretical Gaussian statistical model were used to extrapolate the empirical ROC curve from that point (and to then parametrically measure *A*_*z*_ and/or *d'* = zHR − zFAR), the result would be that *A*_*z*-2AFC_ > *A*_*z*-old/new_ and *d'*_2AFC_ > *d'*_old/new_. More specifically, given the standard assumptions of signal detection theory, it should be the case that *d'*_2AFC_ = (√2) *d'*_old/new_ (Macmillan & Creelman, 2005). This is true even though a model that was cognizant of the procedural difference between the two testing procedures, when fit to the data, would correctly reveal that *d'*_*m*_ is the same for old/new and 2AFC recognition. In other words, it would be the structural aspects of the testing procedure itself (not an underlying difference in *d'*_*m*_ or any other latent variable) that is responsible for the difference in their empirical ROC curves.

The key point is that, for multiple reasons, empirical ROC curves can differ between two conditions even when *d'*_*m*_ is equated. The two conditions might yield different empirical ROCs because (1) despite being equated in terms of latent memory signals (*d'*_*m*_), they differ in terms of a different latent variable (e.g., criterion variability) or (2) despite being equated in terms of *all* latent variables, the format of one testing procedure facilitates empirical performance relative to a different testing format (e.g., 2AFC vs. old/new recognition). Our claim is that policymakers should care only about getting performance on the highest empirical ROC, no matter how one gets there and no matter how the two procedures compare in terms of underlying latent variables.^4^Again, the reason is that the procedure that yields the higher empirical AUC can achieve both a higher HR and a lower FAR than a competing procedure. Theoreticians, by contrast, are interested in understanding the variables that specifically affect underlying latent variables. The better we understand the factors that affect *d'*_*m*_, for example, the better positioned we will be to figure out how to increase it (thereby elevating the empirical ROC, all else being equal).

### The role of latent variables in the ROC controversy

The controversy over ROC analysis in the eyewitness identification literature is largely predicated on the idea that, in prior work, we have claimed the opposite of what we are claiming here (i.e., that the applied implications of ROC analysis come from *d'*_*m*_, not pAUC). For example, in the latest critique of ROC analysis, Smith et al. argued against the following idea, which they attributed to us: “…the procedure that produces superior *underlying discriminability* produces superior applied utility” (Smith et al., 2017, p. 127, emphasis added). Similarly, Lampinen (2016), again citing us, suggested that “One reason one might argue for the use of the ROC approach, over more traditional analyses, is if one believes that area under the ROC curve provides a better index of *underlying memory discriminability*” (Lampinen, 2016, p. 24, emphasis added). Both were referring to what we have here denoted *d'*_*m*_ (i.e., the degree to which the distributions of target and foil memory signals overlap).

Contrary to these claims, we have not argued that the procedure that yields higher *d'*_*m*_ is the procedure that policymakers should prefer. Instead, from the beginning, we have claimed that the procedure that yields higher atheoretical (non-parametric) pAUC is the procedure that policymakers should prefer (e.g., see Mickes et al., 2012, p. 368, where we first explain the problem with relying on a theoretical measure like *d'*). It is, in fact, why Mickes et al. (2012) actually estimated pAUC – not *d'*_*m*_ – from ROC data to make claims about the applied implications of our research comparing simultaneous vs. sequential lineups. Nevertheless, it seems clear that we were understood as having made the exact opposite claim, which is why we have addressed the issue in much greater detail in the present article.

Both Lampinen (2016) and Smith et al. (2017) ran simulations showing that pAUC can differ across eyewitness identification procedures even when *d'*_*m*_ is equated for the two procedures. Both judged that result to be problematic for ROC analysis, but, as explained above, a dissociation between *d'*_*m*_ and pAUC can arise for multiple reasons and is not in any way problematic. We next illustrate that point in more detail by considering an extreme scenario in which the two measures go in *opposite* directions (i.e., when *d'*_*m*_ is higher for Condition A but pAUC is higher for Condition B). To appreciate why the two measures can go in opposite directions without contradiction, it is important to consider how *d'*_*m*_ would actually be estimated from ROC data like those depicted earlier in Table 1. As we will see, fitting a theory-based signal detection model to multiple ROC points to measure latent variables like *d'*_*m*_ and criterion variability is fundamentally different from using a generic signal detection model to extrapolate the empirical ROC curve from a single point in order to parametrically estimate *A*_*z*_.

### Measuring *d'*_*m*_ and criterion variability

To measure *d'*_*m*_ from lineup data, one needs to fit a model that is cognizant of the task demands (e.g., whether the task is a showup, a 2AFC task, a simultaneous lineup, or a sequential lineup) and that can also separate the effects of variability in criterion placement from the effects of variability in memory signals. We describe one such model here for the simultaneous procedure and then consider how to actually fit that model to empirical ROC data.

According to the model presented earlier in Fig. 2, signal detection theory holds that unobservable (latent) memory strength values for targets and foils are distributed according to Gaussian distributions with means of *μ*_*Target*_ and *μ*_*Foil*_, respectively. For a simultaneous lineup, we noted that the simplest decision strategy on both target-present and target-absent trials would be to first identify the photo that generates the strongest signal and to then declare it to be a target-present trial if that signal exceeds a decision criterion (with confidence determined by the highest confidence criterion that is exceeded). This decision rule is usually called the MAX decision rule in perception research (e.g., Eckstein, Thomas, Palmer, & Shimozaki, 2000; Nolte & Jaarsma, 1967; Palmer, Fencsik, Flusberg, Horowitz, & Wolfe, 2011; Palmer, Verghese, & Pavel, 2000; Swensson, 1996; Swensson & Judy, 1981) and is often called the BEST decision rule in eyewitness identification research (Clark, 2003; Clark, Erickson, & Breneman, 2011).^5^

The ability of participants to discriminate between targets and foils is represented by the theoretical distance between the means of the *μ*_*Target*_ and *μ*_*Foil*_ distributions. Assuming an equal-variance model (i.e., *σ*_*Target*_ = *σ*_*Foil*_ = *σ*) the measure of theoretical discriminability is what is usually denoted *d'* and what we here denote *d'*_*m*_ to underscore the fact that we are measuring the degree to which underlying *memory* signals overlap. This model assumes that the two distributions have equal variance, but an unequal-variance model sometimes fits the data better (e.g., Mickes et al., 2017). In that case, the measure of theoretical discriminability would no longer be *d'*_*m*_ but would instead be *d*_*a*_ (Macmillan & Creelman, 2005). The formula for *d*_*a*_ is as follows:

$$ {d}_a=\left({\mu}_{Target}-{\mu}_{Foil}\right)/\surd \left[\frac{1}{2}\left({\sigma^2}_{Target}+{\sigma^2}_{Foil}\right)\right]. $$

When *σ*_*Target*_ = *σ*_*Foil*_, we can replace both by *σ*, and the denominator reduces to √ [½(*σ*^*2*^ + *σ*^*2*^)] = √ *σ*^*2*^ = *σ*. In that case, the formula reduces to the equation for *d'*_*m*_. Thus, the above equation quantifies discriminability for the general case.

Fitting a signal detection model for lineups to data like those shown in Table 1 involves estimating at least six parameters using steps detailed in the Appendix. As described there, fitting the model requires specifying separate likelihood functions for suspect IDs, filler IDs and lineup rejections (No IDs) for target-present and target-absent lineups. These functions can then be used to adjust the six parameters in such a way as to minimize chi-squared deviations between predicted and observed data or to maximize the likelihood of the data. The six parameters consist of the five confidence criteria (the mean locations of the five confidence criteria on the decision axis, *μ*_*C1*_ through *μ*_*C5*_), plus *μ*_*Target*_ (*μ*_*Foil*_ is fixed at 0 and *σ*_*Target*_ and *σ*_*Foil*_ are both fixed at 1 in the equal-variance case). The *σ*_*Target*_ parameter can be allowed to differ from 1 to test whether the ROC data are better fit by an unequal-variance model. If the locations of the confidence criteria are assumed to vary to an appreciable degree across trials or (in the case of aggregated data) across observers, then a seventh parameter, *σ*_*C*_ (the standard deviation of the criterion locations), could be estimated as well. Referring to Fig. 2, setting *σ*_*C*_ > 0 means that the placement of a confidence criterion like *c*_5_ (for example) varies from witness to witness instead of remaining fixed (i.e., instead of *σ*_*C*_ = 0).

When the equal-variance version of the model is fit to the ROC data in Table 1, it yields an estimate of theoretical discriminability in terms of *d'*_*m*_*.* To the extent that the assumptions of the model are accurate, the estimate of underlying discriminability is also accurate. We actually generated the hypothetical data shown in Table 1 using the equal-variance model shown in Fig. 2 (with *d'*_*m*_ set to 1.4 and *σ*_*C*_ set to 0) using a MAX decision rule, so the model would necessarily fit those data well indeed, and *d'*_*m*_ would be estimated to be about 1.4. Critically, had we set *σ*_*C*_ to a value greater than 0, the simulated empirical ROC data would have fallen closer to the line of chance performance. If the model were fit to *those* data, the estimate of *σ*_*C*_ would now be greater than 0, but *d'*_*m*_ would be still estimated to be about 1.4. In other words, there would be a dissociation between pAUC and *d'*_*m*_. We next illustrate this dissociation by considering the even more extreme scenario where the two measures go in opposite directions.

### Can pAUC and *d'*_*m*_ return opposite conclusions?

**Fig. 6:**
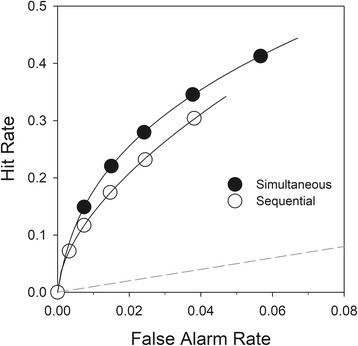
The same receiver operating characteristic (ROC) data as in Fig. 5 except that the smooth curves generated by a theoretical (signal detection) model are drawn through the ROC data points. The dashed line represents chance performance. To generate these data, *d'* was set to 1.4 for the simultaneous procedure and to 1.6 for the sequential procedure. The confidence criteria for the simultaneous lineup ranged from 1.5 (the overall decision criterion, *c*_1_) to 2.5 (the high-confidence decision criterion, *c*_5_). The corresponding confidence criteria for the sequential lineup ranged from 2.0 to 3.0, which captures the widely held view that sequential lineups induce more conservative responding than simultaneous lineups. Finally, criterion variance (*σ*_*C*_) was set to 0 for the simultaneous lineup and to 0.75 for the sequential lineup, which is why the sequential lineup, despite its higher *d'*, yields a lower ROC than the simultaneous lineup

The hypothetical simultaneous lineup data shown in Fig. 6 (filled circles) were generated by the model depicted in Fig. 2 using the MAX decision rule. They are, in fact, the same simultaneous lineup ROC data shown earlier in Figs. 4 and 5, with *d'*_*m*_ = 1.4. Now, however, the ROC points have not been atheoretically connected by straight lines. Instead, a smooth curve has been drawn through the ROC data, and the curve was generated by the model with *d'*_*m*_ = 1.4 and with *σ*_*C*_ = 0. For the sequential lineup, the data were also generated by a model like the one depicted in Fig. 2 but with three important differences described next.

First, *d'*_*m*_ for the sequential lineup was set to the higher value of 1.6. A longstanding theory of why sequential lineups are superior to simultaneous lineups holds that sequential lineups encourage an “absolute” judgment strategy in which each face is individually compared to memory of the perpetrator, whereas simultaneous lineups encourage a “relative” judgment strategy in which the faces in the lineup are judged in relation to each other. The absolute/relative distinction was originally advanced as a theory of response bias, with a relative judgment strategy corresponding to increased pressure to choose someone from the lineup. In other words, a relative judgment strategy was originally construed as a liberal response bias (Wells, 1984). However, if an absolute judgment strategy also decreased the overlap in the memory signals of innocent and guilty suspects for some reason, which is an idea that is sometimes entertained (e.g., Clark et al., 2011), then underlying *d'*_*m*_ would be higher for the sequential procedure. For illustrative purposes, we assume that *d'*_*m*_ is in fact higher for the sequential procedure because it encourages an absolute judgment strategy.

Second, a “first-above-criterion” decision rule was used instead of the MAX decision rule (Kaesler, Semmler, & Dunn, 2017) because the sequential procedure typically stops when the first face is identified. The memory signal associated with that face, which is not necessarily the MAX face in the lineup, determines the level of confidence. If these data were fit by a model to estimate *d'*_*m*_, the model would have to be cognizant of this decision rule. Thus, it would differ from the model outlined in the Appendix and would instead correspond to the model used by Kaesler et al. (2017).

Third, criterion variability was introduced by setting *σ*_*C*_ = 0.75. That is, each confidence criterion was associated with a *mean* location instead of a fixed location, and each had a standard deviation of 0.75. As noted earlier, criterion variability harms empirical discriminability. Thus, although we programmed a *d'* advantage for the sequential procedure (setting *d'*_*m*_ = 1.6 for the sequential lineup and *d'*_*m*_ = 1.4 for the simultaneous lineup), we programmed a criterion variability advantage for the simultaneous procedure (setting *σ*_*C*_ = 0 for the simultaneous lineup and *σ*_*C*_ = 0.75 for the sequential lineup). Criterion variability might be higher for the sequential procedure because instead of making only one decision per lineup, as a witness presented with a simultaneous lineup does, a witness presented with a sequential lineup makes as many as six decisions, with each decision providing an opportunity for the placements of the confidence criteria to change.

For the hypothetical data in Fig. 6, even though underlying theoretical discriminability (*d'*_*m*_) is greater for sequential lineups than it is for simultaneous lineups, the sequential ROC data nevertheless fall closer to the diagonal line of chance performance (i.e., pAUC is lower for the sequential procedure, and parametric *A*_*z*_ would be lower as well). The reason is that criterion variability has a similar effect on the ROC as reducing *d'*_*m*_, which is that is the ROC data are pulled down closer to chance performance (Macmillan & Creelman, 2005). In our example, the programmed advantage of increased theoretical discriminability for the sequential condition is more than counteracted by the programmed disadvantage of increased criterion variability and leads to a lower empirical ROC. The resulting ROC data are such that an AUC measure (whether parametric or non-parametric) would be lower for the sequential procedure. That is, pAUC_SIM_ > pAUC_SEQ_ (as illustrated earlier in Fig. 5 for these same ROC data), despite the fact that, in terms of underlying theoretical discriminability, *d'*_*m*-SEQ_ > *d'*_*m*-SIM_.

In a case like this, which procedure yields higher discriminability? Is one measure right and the other wrong? In truth, both measures are right, but they answer different questions. The *d'*_*m*_ measure is right because the distributions of underlying memory signals in the brains of eyewitnesses are in fact less overlapping for sequential lineups than simultaneous lineups (as might be predicted by a psychological model). Indeed, if the appropriate signal detection models were fit to the two ROC functions in Fig. 6 (i.e., models that were cognizant of the different decision rules used for simultaneous and sequential lineups and that separately estimated *d'*_*m*_ and criterion variability), they would correctly reveal that while criterion variability (*σ*_*C*_) is greater for the sequential procedure, *d'*_*m*_ is also greater for the sequential procedure. If a model predicted the observed difference in *d'*_*m*_ (higher for the sequential procedure in this hypothetical example), the data would support that model even though empirical discriminability (pAUC) goes in the opposite direction.

Nevertheless, the pAUC measure is also right because, in this hypothetical example, eyewitnesses are more likely to correctly sort innocent and guilty suspects into their true categories when simultaneous lineups are used compared to when sequential lineups are used (i.e., pAUC_SIM_ > pAUC_SEQ_). A Gaussian-based parametric measure of AUC would also correctly reveal a simultaneous advantage in terms of empirical discriminability (i.e., *A*_*z*-SIM_ > *A*_*z*-SEQ_). The sequential procedure suffers in this example because the ability to correctly sort innocent and guilty suspects is determined not only by the degree to which the underlying memory signals overlap but also by criterion variability. As a result, in actual practice, eyewitnesses would better distinguish between innocent and guilty suspects using the simultaneous procedure. Thus, in this example, both measures – *d'*_*m*_ and pAUC (or *A*_*z*_) – are correct despite what, superficially, looks like a blatant contradiction.

## Competing theories of underlying discriminability for lineups

Having illustrated the key difference between empirical and theoretical discriminability, we now consider two recently proposed theories of why empirical discriminability (pAUC) differs for different eyewitness identification procedures. The empirical result of interest is that simultaneous lineups yield a higher pAUC than both sequential lineups and showups (Carlson & Carlson, 2014; Dobolyi & Dodson, 2013; Gronlund et al., 2012; Mickes et al., 2012; Neuschatz, Wetmore, Key, Cash, Gronlund & Goodsell, 2016; Wetmore, Neuschatz, Gronlund, Wooten, Goodsell & Carlson, 2015). In 2014, we advanced a theory according to which, compared to sequential lineups, simultaneous lineups help witnesses to notice and to then discount non-diagnostic facial features (namely, the features that are common across the lineup members). By discounting non-diagnostic features, eyewitnesses are better able to focus attention on diagnostic features (Wixted & Mickes, 2014). This is a theory of underlying theoretical discriminability, and it assumes that pAUC is greater for simultaneous than sequential lineups precisely because underlying *d'*_*m*_ is also greater for simultaneous than sequential lineups. That is, according to this theory, there is no dissociation between conclusions based on *d'*_*m*_ and pAUC. The same theory accounts for why simultaneous lineups also yield higher empirical discriminability than showups. In a showup, the test consists of a single face (i.e., the innocent suspect or the guilty suspect presented in isolation), so there is no opportunity to learn about non-diagnostic facial features. In summary, according to this theory, pAUC_SIM_ > pAUC_SEQ_ and pAUC_SIM_ > pAUC_SHOWUP_ because *d'*_*m*-SIM_ > *d'*_*m*-SEQ_ and *d'*_*m*-SIM_ > *d'*_*m*-SHOWUP_, respectively.

According to a competing theory recently proposed by Smith et al. (2017), showups yield the same underlying discriminability as simultaneous lineups (i.e., *d'*_*m*-SIM_ = *d'*_*m*-SHOWUP_), but their empirical ROCs measured in terms of pAUC differ due to other factors. According to this model, different eyewitness identification procedures are differentially susceptible to the deleterious effects of criterion variability. Their simulations showed that, in the absence of criterion variability and with *d'*_*m*_ equated, the two procedures produce comparable empirical ROC curves. However, in the presence of criterion variability (equated across the two procedures), simultaneous lineups yielded higher empirical discriminability (measured by pAUC) than showups. This result is not unlike the difference in AUC produced by old/new and 2AFC recognition tests even when underlying latent variables are equated across testing procedures. In both cases, it is the structural constraints of the testing procedure itself, not a difference in underlying latent variables, that results in a difference in the empirical ROC curves. Smith et al. (2017) did not investigate what their criterion variability theory predicts about simultaneous vs. sequential lineups, so we replicated their simulation and extended it to include the sequential procedure.

**Fig. 7:**
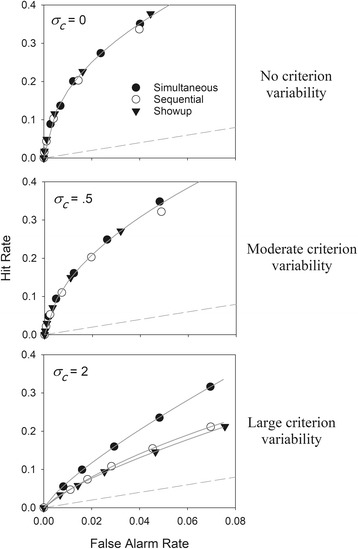
Simulated receiver operating characteristic (ROC) data generated by a simultaneous lineup using the MAX decision rule, a sequential lineup using the “first-above-criterion” decision rule, and a showup. A showup is an old/new recognition memory task in which a single face is presented for an old/new decision. For all three procedures, *d'* was set to 1.4, the overall decision criterion was set to 1.7, and 100,000 simulated trials were run. The top panel shows the simulated results with criterion variability set to 0. The middle panel shows the simulated results with criterion variability set to 0.5. The bottom panel shows the simulated results with criterion variability set to 2.0 (extreme criterion variability). The confidence criteria were programmed to shift in lock step to prevent violations of monotonic order (lowest = 1 to highest = 5). The dashed line represents the line of chance performance

For this simulation, *d'*_*m*_ was set to be equal for all three procedures (*d'*_*m*_ = 1.4 for the simultaneous lineup, the sequential lineup and the showup). In addition, criterion variability (*σ*_*C*_) was also set to be equal for all three procedures. Because the relevant latent variables (*d'*_*m*_ and *σ*_*C*_) were the same for all three procedures, one might expect that all three procedures would yield the same empirical ROC. However, as shown by Smith et al. (2017), and as we have long known in the context of old/new vs. 2AFC, that is not always the case. We simulated three scenarios: no criterion variability, medium criterion variability and large criterion variability. Although *d'*_*m*_ was held constant at 1.4 in each run, the value of *σ*_*C*_ was set to 0 for all three procedures in the first run, to 0.5 for all three procedures in the second run, and to 2.0 (the value used by Smith et al., 2017) for all three procedures in the third run. The results of the simulation are shown in Fig. 7.

In the absence of criterion variability (top panel of Fig. 7, *σ*_*C*_ = 0), the three ROC curves basically fall atop one another, which means that empirical discriminability (as measured by pAUC) is predicted to be the same (or at least very similar) for all three procedures. This is the typical situation in which underlying discriminability and pAUC lead to the same conclusion. When moderate criterion variability is added to the model (middle panel of Fig. 7, *σ*_*C*_ = 0.5), the ROCs for all three procedures move closer to the line of chance performance (i.e., criterion variability harms empirical discriminability), but they all still basically trace out the same ROC curve. However, when a large degree of criterion variability is introduced (lower panel of Fig. 7, *σ*_*C*_ = 2.0), the ROCs for all three procedures drop even closer to the line of chance performance and they now begin to separate from each other. This is true even though underlying discriminability has not changed and is still set to *d'*_*m*_ = 1.4 for all three procedures.

As shown by Smith et al. (2017), and as we replicate here, when *σ*_*C*_ = 2.0, empirical discriminability for the showup procedure is impaired to a greater extent than empirical discriminability for the simultaneous lineup procedure. In addition, as we show here for the first time, empirical discriminability for the sequential lineup procedure is also impaired by criterion variability to about the same degree that the showup procedure is impaired. The sequential lineup suffers from criterion variability because its stopping rule pulls performance down towards the diagonal line of chance performance whenever the criterion is randomly liberal (cf. Rotello & Chen, 2016).

The fact that extreme criterion variability predicts the same outcome that is predicted by the diagnostic feature-detection theory means that there are now two competing theories of underlying latent variables that can explain why simultaneous lineups yield a higher pAUC than showups and sequential lineups. The diagnostic feature-detection theory attributes the difference to a *d'*_*m*_ advantage enjoyed by simultaneous lineups compared to the other two procedures. By contrast, the criterion variability theory assumes that *d'*_*m*_ (and *σ*_*C*_) is equal for the three procedures and that the difference in pAUC arises because the simultaneous procedure is less susceptible to the deleterious effects of criterion variability than showups and sequential lineups. Future research will undoubtedly test the predictions of these competing theories by, for example, comparing how well they can fit empirical ROC data, and the results will help to guide efforts to improve eyewitness identification procedures.

## Conclusion

Here, we advanced the argument that when it comes to informing real-world policy decisions about eyewitness identification procedures, an empirical measure of discriminability (pAUC, or its parametric counterpart, when necessary) takes precedence over a theoretical measure of the degree to which memory signals overlap (*d'*_*m*_). The pAUC measure informs policy because, in terms of empirical reality, the procedure that yields the higher area under the ROC can achieve both a higher HR and a lower FAR than a competing procedure. No theory of underlying memory signals (and no measure of *d'*_*m*_) will change that fact.

The idea that discriminability should be measured using ROC analysis for competing eyewitness identification procedures has proven to be controversial. The controversy is based almost entirely on the mistaken idea that the proponents of ROC analysis believe that a measure underlying theoretical discriminability directly informs policy decisions. However, from the beginning, the proponents of ROC analysis have argued *against* using a theoretical measure of discriminability to inform policy decisions and in favor of using an atheoretical (non-parametric) measure of the area under the empirical ROC curve. In other words, we and others have argued that, just as in many other applied fields, policy in the field of eyewitness identification with regard to competing eyewitness identification procedures is informed by the area under the empirical ROC (not by a theoretical measure of the degree to which distributions of underlying memory signals overlap in the brains of eyewitnesses).

Unlike policymakers, theoreticians seek to measure underlying latent variables like *d'*_*m*_ (the degree to which memory signals overlap) and *σ*_*C*_ (criterion variability). By fitting a signal detection model to ROC data, one can separately estimate these parameters to test the predictions of competing theories. Two theories that have been proposed in this regard are the diagnostic feature-detection theory (Wixted & Mickes, 2014) and criterion variability theory (Smith et al., 2017). Both theories predict that pAUC for simultaneous lineups should exceed that for sequential lineups and showups, but for different reasons. The diagnostic feature-detection theory attributes the pAUC effect to a higher *d'*_*m*_ associated with the simultaneous procedure. The criterion variability theory instead attributes the pAUC effect to the fact that the simultaneous procedure is less susceptible to the deleterious effects of extreme criterion variability.

Notably, the architects of the criterion variability theory include the creators of, and the staunchest proponents of, the sequential lineup procedure. The fact that their new theory predicts that simultaneous lineups should be diagnostically superior to sequential lineups in terms of empirical discriminability suggests that a convergence of views may be developing despite an apparent controversy over ROC analysis. According to both theoretical accounts proposed this far (the diagnostic feature-detection theory and the criterion-variability theory), and according to the relevant empirical ROC data, simultaneous lineups are diagnostically superior to sequential lineups in an applied sense (pAUC_SIM_ > pAUC_SEQ_). Viewed in this light, the “controversy” over ROC analysis of lineup performance actually consists of a normal scientific debate about which theory of underlying latent variables better accounts for the empirical data. Critically, the resolution of that debate will have no bearing on the policy implications of ROC analysis. The policy implications are derived from an empirical measure of discriminability (pAUC), which is based on the rate at which innocent and guilty *suspects* (not foils, in the case of lineups) are identified using a particular eyewitness identification procedure. There is no controversy over the claim that pAUC for simultaneous lineups is, in every study conducted thus far, greater than or equal to pAUC for both sequential lineups and showups.

## Appendix

The likelihood functions for fitting a signal detection model to data from a fair lineup can be worked out by specifying the joint probabilities of the events that result in a given outcome (suspect ID, filler ID, or no ID). As an example, consider the probability of identifying the guilty target with memory strength *x*_1_ from a target-present lineup. There is (1) some probability of observing a particular memory strength of the target, *x*_1_, (2) some probability that *x*_1_ will be the highest (MAX) memory strength of the lineup members and (3) some probability, *f(***x**)*,* that the decision variable will exceed the decision criterion (where **x** represents the vector of memory signals associated with the faces in the lineup). The joint probability of those events is the probability that the target will be identified from a target-present lineup. For the Independent Observations model considered in the main text, the decision variable, *f(***x***),* is *x*_1_ itself (i.e., the untransformed MAX memory signal). Here, we describe how to write the likelihood function for that probability and then describe the similar approach used to write the likelihood functions for the probability of observing a filler ID from a target-present and then from a target-absent lineup.

### Probability of observing a target from a target-present lineup

Assuming a standard equal-variance signal detection model, the probability of observing target memory strength *x*_*1*_ (event 1) is given by a Gaussian distribution with mean, *μ*_*1*_ = *μ*_*Target*_ and variance *σ*_1_^*2*^ = *σ*^*2*^_*Target*_:

1 $$ P\left({x}_1\right)=\frac{1}{\sqrt{2\pi {\sigma}_1^2}}{e}^{-{\left({x}_1-{\mu}_1\right)}^2/\left(2{\sigma}_1^2\right)}. $$

The probability that *x*_1_ is greater than the memory strength of a filler *j* is obtained by integrating a Gaussian distribution with mean *μ*_*j*_ = *μ*_*Foil*_ and variance *σ*_*j*_^*2*^ = *σ*^*2*^_*Foil*_ from − ∞ to *x*_1_:

2 $$ \frac{1}{\sqrt{2\pi {\sigma}^2}}{\int}_{-\infty}^{x1}{e}^{-{\left({x}_j-{\mu}_j\right)}^2/\left(2{\sigma}_j^2\right)}{dx}_j=\Phi \left(\frac{x_1-{\mu}_j}{\sigma_j}\right), $$

where Φ is the standard cumulative normal distribution. Thus, the probability that a given *x*_1_ is greater than the value of *all* foils in a lineup of size *k* is:

3 $$ P\left({x}_2\dots k<\left.{x}_1\right|{x}_1\right)=\prod \limits_{j=2}^k\Phi \left(\frac{x_1-{\mu}_j}{\sigma_j}\right). $$

And the probability that *x*_1_ exceeds the decision criterion, *c*, is equal to 1 minus the probability that *x*_1_ falls below c:

4 $$ P\left({x}_1\right.>\left.c\right|{x}_2\dots {x}_k<{x}_1=1-\Phi \left(\frac{c-{x}_1}{\sigma_c}\right), $$

where *σ*_*c*_ is the standard deviation of the criterion placement (*σ*_*c*_ = 0 if no criterion variability is assumed). Thus, the probability of observing *x*_1_* and* the probability that *x*_1_ is greater than the value of all lures in a lineup of size *k and* the probability that *x*_1_ exceeds the decision criterion, integrated over all possible *x*_1_ (i.e., over all possible target memory-strength values), is given by Eq. 1 *×* Eq. 3 *×* Eq. 4 (integrated from − ∞ to + ∞):

5 $$ \frac{1}{\sqrt{2{\pi \sigma}^2}}{\int}_{-\infty}^{+\infty }{e}^{-{\left({x}_1-{\mu}_1\right)}^2/\left(2{\sigma}_1^2\right)}\ \prod \limits_{j=2}^k\Phi \left(\frac{x_1-{\mu}_j}{\sigma_j}\right)\left[1-\Phi \left(\frac{c-{x}_1}{\sigma_c}\right)\right]{dx}_1. $$

Again, this is the likelihood of observing a filler ID from a target-present lineup. In MATLAB, the code for this function could be written as:

$$ \mathrm{f}=@\left(\mathrm{x}\right)\ \mathrm{normpdf}\left(\mathrm{x},\mathrm{mu}\_\mathrm{t},\mathrm{sigma}\_\mathrm{t}\right).\ast \mathrm{normcdf}\left(\mathrm{x},\mathrm{mu}\_\mathrm{d},\mathrm{sigma}\_\mathrm{d}\right).\hat{\mkern6mu} \left(\mathrm{k}-1\right).\ast \Big(1-\mathrm{normcdf}\left(\mathrm{c},\mathrm{x},\mathrm{sigma}\_\mathrm{c}\right), $$

where mu_t = *μ*_*Target*_, mu_d = *μ*_*Foil*_, sigma_t = *σ*_*Target*_, sigma_d = *σ*_*Foil*_, *k* is lineup size, *c* is the confidence criterion and sigma_c = *σ*_*c*_. The parameters *μ*_*Target*_ and *σ*_*Target*_ are estimated by the fitting procedure, whereas *μ*_*Foil*_ and *σ*_*Foil*_ are set to 0 and 1, respectively. This function corresponds to the probability of observing a target ID from a target-present lineup made with a particular level of confidence associated with criterion, *c*. If there are five confidence criteria for making a positive ID (as in Fig. 2), there would be five separate confidence parameters to inset into this equation (*c*_1_ through *c*_5_). Integrating this function from − ∞ to + ∞ yields the probability of a target ID with a particular level of confidence from a target-present lineup, p_t, which can be computed in MATLAB using:

$$ \mathrm{p}\_\mathrm{t}=\mathrm{integral}\left(@\left(\mathrm{x}\right)\ \mathrm{f}\left(\mathrm{x}\right),-15,15\right). $$

### Probability of observing a filler (i.e., a foil) from a target-present lineup

The second likelihood function specifies the likelihood of observing a filler ID from a target-present lineup. The probability of observing filler memory strength *x*_1_ from a target-present lineup is given by Eq. 1 with mean, *μ*_1_ = *μ*_*Foil*_ and variance *σ*_1_^*2*^ = *σ*^*2*^_*Foil*_. The probability that *x*_1_ is greater than the memory strength of another filler *j* in the lineup is obtained by integrating a Gaussian distribution with mean *μ*_*j*_ = *μ*_*Foil*_ and variance *σ*_*j*_^*2*^ = *σ*^*2*^_*Foil*_ from − ∞ to *x*_1_, using Eq. 2. The probability that *x*_1_ is also greater than the memory strength of the target is obtained by integrating a Gaussian distribution with mean *μ*_*j*_ = *μ*_*Target*_ and variance *σ*_*j*_^*2*^ = *σ*^*2*^_*Target*_ from − ∞ to *x*_1_, again using Eq. 2. Thus, the probability that a given *x*_1_ is greater than the value of all the *k* − 2 fillers in a lineup of size *k* is:

$$ P\left({x}_2\dots k<{x}_1|{x}_1\right)=\left[\prod \limits_{j=2}^{k-1}\Phi \left(\frac{x_1-{\mu}_{Distractor}}{\sigma_{Distractor}}\right)\right]\ \Phi \left(\frac{x_1-{\mu}_{Target}}{\sigma_{Target}}\right). $$

And the probability that *x*_1_ exceeds the decision criterion, *c*, is equal to 1 minus the probability that *x*_1_ falls below c:

$$ P\left({x}_1>c\ \right|{x}_2\dots {x}_k<{x}_1=1-\Phi \left(\frac{c-{x}_1}{\sigma_c}\right). $$

Thus, the probability of observing *x*_1_*and* the probability that *x*_1_ is greater than the value of all foils in a lineup of size *k and* the probability that *x*_1_ exceeds the decision criterion, integrated over all possible *x*_*i*_ (i.e., over all possible memory-strength values for a filler) is given by:

$$ \frac{1}{\sqrt{2\pi {\sigma}^2}}{\int}_{-\infty}^{+\infty }{e}^{-{\left({x}_1-{\mu}_1\right)}^2/\left(2{\sigma}_1^2\right)}\ \prod \limits_{j=2}^{k-1}\Phi \left(\frac{x_1-{\mu}_1}{\sigma_1}\right)\Phi \left(\frac{x_1-{\mu}_{Target}}{\sigma_{Target}}\right)\ \left[1-\Phi \left(\frac{c-{x}_1}{\sigma_c}\right)\right]d{x}_1. $$

Again, this is the likelihood of observing a filler ID from a target-present lineup.

### Probability of observing a filler (i.e., a foil) from a fair target-absent lineup

The third and final likelihood function specifies the likelihood of observing a filler ID from a target-absent lineup. The probability of observing filler memory strength *x*_1_ from a target-present lineup is given by Eq. 1 with mean, *μ*_1_ = *μ*_*Foil*_ and variance *σ*_1_^*2*^ = *σ*^*2*^_*Foil*_. The probability that *x*_1_ is greater than the memory strength of another filler *j* in the lineup is obtained by integrating a Gaussian distribution with mean *μ*_*j*_ = *μ*_*Foil*_ and variance *σ*_*j*_^*2*^ = *σ*^*2*^_*Foil*_ from − ∞ to *x*_1_, using Eq. 2. Thus, the probability that a given *x*_1_ is greater than the value of all of the other *k* – 1 fillers in a lineup of size *k* is:

$$ P\left({x}_2\dots k<{x}_1|{x}_1\right)=\prod \limits_{j=2}^k\Phi \left(\frac{x_1-{\mu}_1}{\sigma_1}\right). $$

And the probability that *x*_1_ exceeds the decision criterion, *c*, is equal to 1 minus the probability that *x*_*1*_ falls below c:

$$ P\left({x}_1>c\ \right|{x}_2\dots {x}_k<{x}_1=1-\Phi \left(\frac{c-{x}_1}{\sigma_c}\right). $$

Thus, the probability of observing *x*_1_*and* the probability that *x*_1_ is greater than the value of all other foils in a lineup of size *k*, integrated over all possible *x*_*i*_ (i.e., over all possible memory-strength values for a filler) is given by:

$$ \frac{1}{\sqrt{2\pi {\sigma}^2}}{\int}_{-\infty}^{+\infty }{e}^{-{\left({x}_1-{\mu}_1\right)}^2/\left(2{\sigma}_1^2\right)}\ \prod \limits_{j=2}^k\Phi \left(\frac{x_1-{\mu}_1}{\sigma_1}\right)\ \left[1-\Phi \left(\frac{c-{x}_1}{\sigma_c}\right)\right]d{x}_1. $$

Again, this is the likelihood of observing a filler ID from a target-absent lineup.

## Footnotes

1 In a fair lineup, in which no one is designated to serve as the innocent suspect (as in Table 1), the overall target-absent filler ID rate is linearly related to the innocent-suspect false ID rate (i.e., FAR is equal to one sixth times the filler ID rate). Thus, when comparing two lineup procedures, the target-absent filler ID rate can be used in place of FAR.

2 An alternative view is that ROC analysis is flawed because one must also consider filler IDs in order to decide which of the two lineup procedures was diagnostically superior. However, to date, no example has been proffered according to which pAUC is higher for Procedure A in a given range but Procedure B should nevertheless be recommended to policymakers because of how the two procedures differ with respect to filler IDs.

3 In this simplest case, confidence in the chosen item would be based solely on the strength of the memory signal generated by that item. Alternatively, the subject might create a new psychological variable by subtracting the familiarity of one item from the other, and the recognition decision (and confidence) might be based on that transformed variable. For *this* latent variable, *d’*_2AFC_ > *d’*_old/new_.

4 The only exception would be when implementation costs for the procedure yielding the higher ROC exceed the implementation costs of the procedure that yields the lower ROC.

5 Macmillan and Creelman (2005) referred to the MAX decision rule as the Independent Observations model. Other signal detection models applied to lineups assume that the decision is based on a transformed memory signal, such as the sum of the memory signals associated with the faces in the lineup (Duncan, 2006), but we consider only the simpler Independent Observations signal detection model here.
